# The State of the Art of Lethal Oviposition Trap-Based Mass Interventions for Arboviral Control

**DOI:** 10.3390/insects8010005

**Published:** 2017-01-08

**Authors:** Brian J. Johnson, Scott A. Ritchie, Dina M. Fonseca

**Affiliations:** 1 College of Public Health, Medical and Veterinary Sciences, James Cook University, McGregor Rd., Cairns, QLD 4878, Australia; scott.ritchie@jcu.edu.au; 2 Australian Institute of Tropical Health and Medicine, James Cook University, P.O. Box 6811, Cairns, QLD 4870, Australia; 3 Center for Vector Biology, Rutgers University, 180 Jones Ave., New Brunswick, NJ 08901, USA; dinafons@rutgers.edu

**Keywords:** urban, invasive, *Aedes*, ovitrap, vector control, dengue, Zika, community engagement

## Abstract

The intensifying expansion of arboviruses highlights the need for effective invasive *Aedes* control. While mass-trapping interventions have long been discredited as inefficient compared to insecticide applications, increasing levels of insecticide resistance, and the development of simple affordable traps that target and kill gravid female mosquitoes, show great promise. We summarize the methodologies and outcomes of recent lethal oviposition trap-based mass interventions for suppression of urban *Aedes* and their associated diseases. The evidence supports the recommendation of mass deployments of oviposition traps to suppress populations of invasive *Aedes*, although better measures of the effects on disease control are needed. Strategies associated with successful mass-trap deployments include: (1) high coverage (>80%) of the residential areas; (2) pre-intervention and/or parallel source reduction campaigns; (3) direct involvement of community members for economic long-term sustainability; and (4) use of new-generation larger traps (Autocidal Gravid Ovitrap, AGO; Gravid *Aedes* Trap, GAT) to outcompete remaining water-holding containers. While to the best of our knowledge all published studies so far have been on *Ae. aegypti* in resource-poor or tropical settings, we propose that mass deployment of lethal oviposition traps can be used for focused cost-effective control of temperate *Ae. albopictus* pre-empting arboviral epidemics and increasing participation of residents in urban mosquito control.

## 1. Introduction

The continued global scourge of mosquito-borne dengue fever [1], the recent emergence and explosive spread of chikungunya around the world [2], of Zika fever in South and Central America and, most importantly, its association with fetal microcephaly [3], have provided renewed impetus for the development of effective urban mosquito control. The primary vectors of emerging arboviruses are invasive *Aedes aegypti* and *Aedes albopictus* that thrive in private backyards and/or inside the residence [4,5], limiting the efficacy of area-wide mosquito control approaches [6]. Rising insecticide resistance in *Aedes* [7] often leads to failed control and the short-lived efficacy of adulticides (often only a couple of days) requires frequent applications [8,9]. Therefore, although models often support the use of peri-domestic insecticide space spraying to control dengue, and now Zika, there has been little to no epidemiological evidence that such costly and time consuming control strategies are effective [10].

The evidence is strong for the need for novel control methods, ideally those circumventing the use of insecticides, and that are cheap, allowing for deployment in resource-poor areas and/or at the large scale needed for eradication, a viable aim when addressing invasive species limited by their association to humans and human-modified environments [11,12]. A standard approach in pest control is to exploit “weak links”, critical needs of the pest that may be targeted for control [13], such as the characteristic behavior of urban *Aedes* females to lay eggs in artificial (man-made) water-holding containers [14,15,16]. Of note, in temperate regions, where with some exceptions [17,18] *Aedes*-vectored viruses are still a worry and not a panic, public health campaigns employ residents to empty or remove any water-holding containers from their yards [19]. Even when such advice is supported by information campaigns, however, there has been a lack of entomological data showing that residents on their own can significantly reduce *Aedes* habitat from their properties [20], but see [21]. More importantly, however, there has been scarce evidence that targeting just immature stages has a real impact on adult populations [22], possibly because there is such high baseline immature mortality due to the transient nature of the small containers exploited by invasive *Aedes* [23] and density-dependent regulation due to limited food in containers [24].

**Figure 1:**
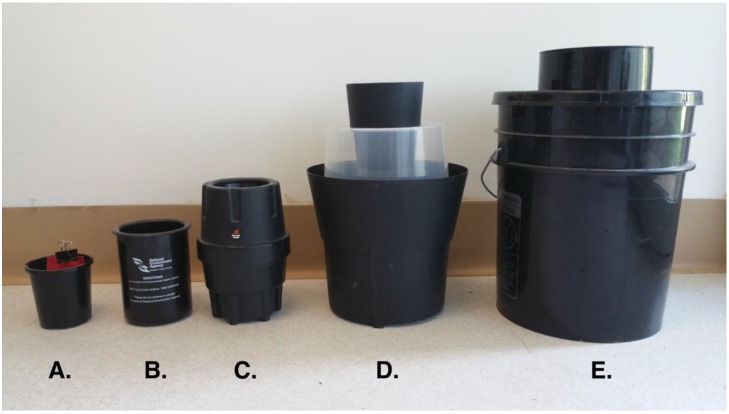
Common ovitraps used in recent mass-trapping campaigns: (**A**) standard (500 mL) lethal ovitrap (LO); (**B**) National Environmental Agency Singapore sticky ovitrap (SO); (**C**) MosquiTRAP sticky ovitrap (SO); (**D**) Biogents Gravid *Aedes* Trap (GAT); and (**E**) Centers for Disease Control (CDC) Autocidal Gravid Ovitrap (AGO).

With the goal of developing new interventions aimed at reducing both adult female mosquitoes and their future offspring, researchers have developed lethal oviposition traps, which are “lure and kill” traps that lure ovipositing females with an attractive infusion and kill them when they attempt to lay eggs (Figure 1). Importantly, these traps target a critical epidemiological stage: gravid female mosquitoes that are more likely to be infected with a vector-borne pathogen than the general adult population, since they have had contact with blood. Females are usually killed quickly, thus killing their progeny, leading to population reductions—larvae or adults that may develop from dropped eggs are killed by residual insecticide or trapped and drowned. Of note, likely a consequence of the high container turnover rate, as well as the need to avoid high-competition environments for the immature, female *Ae. aegypti* and *Ae. albopictus* are “bet-hedgers” that employ strategies to minimize the risk of total reproductive failure, or the loss of all eggs, over space and time by laying their eggs among several oviposition sources [25,26]. This “skip-oviposition” strategy [27,28] means the attractiveness of the trap relative to alternative water-holding containers is critical for control effectiveness. Of note, temperate populations of *Ae. albopictus* allow diapausing eggs to accumulate in the fall [26], possibly to maximize the chance of successful emergence in the spring, a strategy that can be easily exploited for control.

The first modern example of a lethal trap for mosquito control can be credited to Lok et al. [29] and consisted of a black, water-filled cylindrical container with a flotation device made up of a wire mesh and two wooden paddles. Although ovipositing females were not killed, after eggs laid on the wooden paddles hatched, larvae developed in the water under the wire mesh and emerging adults trapped under the wire mesh drowned. While the trap was largely successful at eliminating *Ae. aegypti* from the Singapore International Airport in the late 1970s [29], it was prone to mechanical failure and, as mentioned, did not kill adult females. Thus, the next step in trap evolution was the development of the lethal ovitrap (LO, Figure 1a), the first lethal oviposition trap. LOs are commonly comprised of a small black plastic cup (400–700 mL) containing an insecticide treated ovistrip that kills ovipositing females attracted by the hay and water infusion [30,31,32]. LOs can be very cheap (<US$1) and therefore hundreds of traps can be deployed simultaneously across many individual residences to achieve high coverage. A drawback of LOs, however, is that they are ineffective against insecticide-resistant individuals [30,32,33,34]. To overcome the problem imposed by insecticide resistance, several researchers developed a sticky ovitrap (SO; Figure 1b,c) [35,36,37,38]. SOs use the same “lure and kill” strategy of LOs, but instead of insecticide treated ovistrips, they contain an adhesive strip that captures and kills ovipositing females. The added benefit of SOs is that they can be used for mosquito surveillance or to determine viral infection rates in local mosquito populations. 

But a trade-off of the low price of the original LOs and SOs is their small size and consequently high evaporation rates, which mandates short maintenance intervals (at least once a week depending on rainfall). A larger trap, besides holding more attractive infusion [39], allows for longer maintenance intervals and also provides a more conspicuous visual target for gravid females searching for suitable oviposition sites by a greater release of infusion and olfactory attraction. When dealing with females that “skip-oviposit” and the almost impossible task of removing all other sources of standing water where a female may choose to lay eggs [14,16], it makes sense to stack the odds towards deploying the most attractive lethal oviposition trap and kill the ovipositing females at their first try. While urban *Aedes* will enter small openings to gain access to water in hard-to-find (cryptic) areas [26,40], they typically will first lay eggs in open containers of easy access, if those are present [26]. Of note, one of the foreseeable drawbacks of source reduction without providing alternative oviposition substrates (such as the traps) is that cryptic habitats, which are harder to find, may become the primary larval production sources, further complicating *Aedes* control [16,41].

To harness the potential benefits derived from a larger ovitrap, researchers have recently developed the Centers for Disease Control and Prevention Autocidal Gravid Ovitrap (AGO; Figure 1e; [39]) and the Gravid *Aedes* Trap (GAT; Figure 1d; [42]). The AGO is a large (19 L) black bucket with a relatively large opening (3.8 L black cylindrical entrance) in which an adhesive panel is placed and is baited with 10 L of water-hay infusion. In contrast to the solid black design of the AGO, the GAT employs a compartmentalized design with a black bottom bucket with 3 L of water-hay infusion (or other types as needed) and a translucent middle collection chamber with a black entry funnel. The translucent collection chamber helps retain captured mosquitoes by exploiting their “fly to the light” behavior once they enter the trap. While the recommended killing agent in the GAT is the application of a long-lasting surface spray to inside of the collection chamber, it has recently been demonstrated that using a hanging adhesive panel or applying edible canola oil to the inside of the collection chamber are effective non-insecticide killing agents [43].

Although the traps differ in design, both the AGO and GAT have achieved the desired effect of outperforming standard ovitraps and other lethal autocidal ovitraps in attractiveness to *Aedes*. Field trials in Puerto Rico have demonstrated that the AGO captured more gravid females and provided greater sensitivity (number of traps positive for *Ae. aegypti*) than conventional ovitraps [39], whereas in field trials in Northern Australia, GATs collected 2–4 times more female *Ae. aegypti* than two variations of SOs, the MosquiTRAP and the double sticky ovitrap [44]. Results from the AGO and GAT trials support the notion that a larger size and greater amount of infusion increases trap efficacy. The flexibility of the GAT allowing the use of a variety of killing agents—including edible canola oil—makes it customizable and acceptable by residents that want to develop community-based *Aedes* control [45].

Because LOs, SOs and large AGOs and GATs all satisfy many of the requirements for sustainable vector control (i.e., simple, cheap, target adult females), they make ideal candidates for mass-trapping interventions. Here we summarize recent (2000–2016) mass-deployments of lethal oviposition traps. We then provide a summary/discussion of best operating procedures and discuss limitations, opportunities and future directions.

## 2. Mass Lethal Ovitrapping Interventions 

### 2.1. LO Mass-Trapping in Brazil and Thailand

Two of the earliest attempts to reduce female *Ae. aegypti* populations using simple LOs were conducted by Perich et al. [30] in Brazil, and Sithiprasasna et al. [33] in Thailand. Both studies involved the placement of 10 LOs/residence for 12 weeks [30] or 30 weeks [33], and the sampling of 30 houses per intervention neighbourhood. Both studies used the same LOs comprised of a small, 473 mL black plastic cup baited with 10% hay (w/v) infusion and containing a 11 × 2.5 cm ovistrip treated with deltamethrin, a pyrethroid. Neither study provided information on the proportion of houses with LOs in the intervention areas or implemented any concurrent vector control (no space spraying, source reduction, or larviciding). Despite the lack of additional control, these wide-scale deployments of LOs resulted in a >40% reduction in adult female abundance in at least one site, a 49%–80% reduction in containers positive for *Ae. aegypti* larvae, and a 56%–97% reduction in the mean number of pupae per house. Despite these successes, the observed impact on viral transmission risk, particularly in the Sithiprasasna et al. [33] study, was lower than desired. The failure was attributed to immigration of *Ae. aegypti* from adjacent areas, reduced lethality of the ovistrip after field exposure, and competition from alternative breeding sites. However, particularly because no source reduction or other complementary interventions were implemented, these standalone LO results provide compelling support for multi-trap per residence LO-based interventions.

### 2.2. LO Mass-Trapping in Australia

Despite the relative success of the early LO mass-trapping interventions—or maybe because of it—there was a lack of adoption of LO-based control interventions (at least published in the peer-reviewed literature) until those conducted by Rapley et al. [46] in Cairns, Australia. In two studies in separate areas of Cairns, 1.2 L LOs were deployed four per residence with 71%–93% coverage within the intervention areas and both were preceded by area-wide larval control interventions. In the first deployment, the trap buckets were made of durable plastic, while in a second deployment they were made of biodegradable material. The hay infusion was 0.5 g alfalfa/1 L water and each LO contained a 13.5 × 5 cm flannel ovistrip treated with bifenthrin, another pyrethroid. Larval control interventions included a source reduction campaign and the treatment of non-removable containers with S-methoprene pellets, a long-lasting insect growth regulator [47,48]. The primary results were an 87% reduction in female collections during the mass trap deployment in concurrently monitored SOs during the wet season, but, unfortunately, there were no observed reductions during the following dry season. Likewise, the biodegradable LO intervention achieved only an unspecified reduction in adult female collections in concurrently monitored SOs in one of three sites. Despite the generally low efficacy of both LO interventions, the study achieved high public acceptability [49] and the lack of significant adult control was attributed to the relatively short duration of the interventions (four weeks). Of note, despite pre-intervention, large-scale source reductions and additional larval control efforts before both experiments, they were not as effective as those reported by Perich et al. [30] or Sithiprasasna et al. [33]. Based on their results, the authors concluded that LOs could be an effective component of a dengue control strategy, particularly when coupled with pre-intervention larval control interventions. While not formally quantified, this conclusion has been largely supported by the absence of an explosive dengue outbreak on Thursday Island in the Torres Strait, Australia, where intervention strategies involving source reduction, limited indoor residual spraying, larviciding, and the widespread use of LOs and SOs to suppress local *Ae. aegypti* populations have been implemented [50].

### 2.3. Mass Deployment of SOs in Brazil

As mentioned, the small SOs were developed to circumvent the problems of insecticide resistance and to allow the simultaneous surveillance of adult gravid mosquitoes by capturing them on a sticky panel as they enter the trap. Interestingly, although SOs were developed over 10 years ago [35], only one study to date has assessed their utility in a mass-trapping intervention. The study was conducted by Degener et al. [51] in Manaus, state of Amazonas, Brazil, and used the commercially available MosquiTRAP (Ecovec Ltd., Belo Horizonte, MG, Brazil; Figure 1c) [52]. The MosquiTRAP consists of a matte black container (16 cm high × 11 cm diameter) containing approximately 280 mL of water, a synthetic AtrAedes^®^ (Ecovec Ltd.) oviposition attractant and a removable sticky card. Importantly, Degener et al. [51] monitored epidemiological outcomes by monitoring dengue virus (DENV) IgM-seropositivity of residents in the intervention and control clusters during the last two months of the intervention. The study involved a matched pair cluster design (three treatment and three control areas) during which three MosquiTRAPs were placed at each participating residence in the treatment clusters. Traps in the same household were positioned at least 5 m apart from each other, preferentially in different environments (e.g., veranda, yard, laundry area). In total, 51.1% of available households participated in the mass-trapping effort in the intervention clusters and the intervention lasted 17 months.

Unfortunately, despite high household participation and the deployment of multiple traps at individual residences, mass-deployment of MosquiTRAPs failed to reduce adult *Ae. aegypti* abundance and serological data indicated that recent dengue infections were equally frequent in the intervention and control areas. The failure of the MosquiTRAP mass-trapping intervention may have resulted from its previously quantified poor performance relative to standard ovitraps (78.3% ovitraps positive for eggs vs. 46.4% MosquiTRAPs positive for adult females, *p* < 005, [53]) and the relatively low number of traps deployed per residence (three/house). More importantly, however, it is likely that the lack of pre-intervention source reduction campaigns also contributed to the poor performance of the relatively small MosquiTRAP due to competition from other water sources.

## 3. Large-Scale Mass-Trapping Interventions

### 3.1. Multi-Year Trapping Intervention in Brazil

Although the above studies provided evidence supporting mass-trapping intervention strategies targeting ovipositing females, the studies were relatively small in scale (<200 houses) making it hard to assess the sustainability of large-scale interventions. This was first addressed in a study performed by Regis et al. [54] during which 8400 small (2 L) ovitraps were deployed over a two-year intervention period, during which five ovitraps were placed at each participating residence. Unlike the mechanical suppression of adults in Lok et al. [29], control in the Regis et al. [54] trap intervention relied on the physical collection and destruction of eggs or treatment of containers with *Bacillus thuringiensis israelensis* (Bti) to kill any larvae that developed. The decision not to rely on insecticide treated ovistrips was most likely based on the presence of insecticide resistance in local *Ae. aegypti* populations in the state of Pernambuco, Brazil, in which the study was conducted [55]. To make such a large-scale effort affordable, the LOs were made from recycled 2 L bottles that were painted black, allowing traps to be produced at a cost of US$0.6 each. To overcome the problem of keeping track of thousands of ovitraps, the research team coordinated their efforts with municipal health departments, which were allowed to design their own intervention plans according to the local characteristics and resources available, and operated a network of georeferenced sentinel ovitraps to monitor the impact of the interventions. Altogether, control activities centered around: (1) mechanical mass destruction through incineration of eggs laid on ovistrips; (2) indoor systematic removal of adults using aspirators, targeting places considered highly important for virus transmission, such as health units, schools and premises located within hotspots of mosquito density; (3) the addition of larvivorous “piabas” fish added to nearly 7000 cisterns, the primary local water reservoirs; and (4) educating the public about the importance of source reduction through public exhibition, radio, television, banners, posters and leaflets. This stratified strategy resulted in decreases of 90% and 77% in egg density in sentinel ovitraps in the intervention areas relative to paired controls after two years of sustained pressure. Overall, control efforts destroyed more than 8,000,000 eggs between the two sites and at least 3200 adult females by aspiration. These results demonstrate that wide-scale interventions can be successful if enough traps can be deployed, which is only likely if expertise and resources are shared between researchers and municipal public health agencies, and effective public engagement campaigns can be implemented. Unfortunately, because of the diversity of approaches (source reduction, ovitraps, adult aspiration, and use of larvivorous fish), the authors were unable to determine the level of impact of each individual intervention. Most regrettably, however, the impact of such a wide-scale mass-trapping intervention on disease transmission remains unknown since the authors did not measure epidemiological variables such as mosquito infection rate or seropositivity rates in local residents.

### 3.2. Large Autocidal Gravid Ovitrap-Based success in Puerto Rico

Encouraged by the potential impact of large-scale trapping, a series of interventions using mass deployments of large AGOs were conducted in Puerto Rico from 2011 to 2014 [56,57] to exploit the greater visual and olfactory attraction afforded by large AGOs (Figure 1e) in a mass-trapping intervention. The studies included two intervention zones, La Margarita (2011–2014) and Villodas (2013–2014), each with a paired control area. The experimental design for both studies involved placing three AGOs per house at 81% [56] and 85% [57] of the houses in each intervention area. The researchers implemented a two-month service interval to take advantage of the extended activity of the large AGOs (long-lasting stickiness of the glue panel and large water volume and closed design reduces likelihood the traps will dry) to limit the amount of staff and resources required to service the large number of traps deployed (>700 traps). Because of the long service interval, adult populations were monitored using a combination of BG-Sentinel (BGS) traps and sentinel AGO traps distributed throughout the intervention and control areas. In addition to the trap intervention, pre-intervention source reduction, larviciding, and oviciding campaigns were conducted in both the intervention and control areas. Source reduction consisted of the removal of all containers as allowed by residents. The larvicide Natular (spinosad) was applied to containers that could not be removed and which water was not for animal or human consumption. The inner walls of containers that could not be removed were also brushed and rinsed to remove *Ae. aegypti* eggs.

The pre-intervention source reduction and larviciding campaigns combined with the wide-scale distribution of large AGOs proved highly successful. Over the course of the first study (2011–2012), the La Margarita (812–1050 traps) intervention area experienced a 53% and 70% reduction in weekly BGS and sentinel AGO collections, respectively, relative to the control area. During the second study, the La Margarita (2012–2014; 793 traps) and Villodas (2013–2014; 570 traps) intervention areas experienced a 79% and 88% reduction in weekly sentinel AGO collections, respectively, relative to their paired control areas. To date, these two studies provide the greatest support for large-scale mass-trapping interventions as a means of controlling urban *Aedes*. The success of these interventions is most likely due to the careful large-scale pre-intervention source reduction effort coupled with the greater visual and olfactory attraction of the large AGO compared to smaller LOs and SOs, with the added benefit of the long service intervals of large AGOs enabling greater deployment scale and sustainability. 

### 3.3. Epidemiological Support for Mass-Trapping Interventions in Puerto Rico

Although the large AGO interventions were highly successful, the lack of epidemiological impact continued to impede the true assessment of the effectiveness of large-scale AGO mass-trapping interventions for control *Aedes*-borne viruses. However, the chikungunya virus (CHIKV) epidemic in the Caribbean from 2014 to 2015 allowed the quantification of the efficacy of a large AGO-based mass-deployment for arboviral control. From early 2014 to the end of 2015, there were approximately 29,000 confirmed chikungunya cases in Puerto Rico, which by fortunate happenstance, overlapped with the AGO mass-trapping interventions in Puerto Rico. As mentioned, the interventions resulted in a ten-fold reduction in *Ae. aegypti* female densities (~1 per trap/week in intervention sites vs. ~10 per trap/week in control sites) during the 2014–2015 chikungunya epidemic.

To assess the effect of the areawide deployment of AGO traps on chikungunya prevalence, Lorenzi et al. [58] conducted a stratified random serosurvey of 620 households from intervention and non-intervention communities, representing 28.5% of the residents of the communities participating in the AGO field trial sites of Barrera et al. [57]. Serum specimens were tested by immunoglobulin G (IgG) enzyme-linked immunosorbent assay to detect evidence of recent chikungunya virus infection. The authors reasoned that the prevalence of CHIKV IgG antibody after the introduction of chikungunya in a population without previous chikungunya virus exposure provided a valid estimate of chikungunya virus incidence in residents of these communities. After adjustment for sample design, the serosurvey revealed that the proportion of CHIKV IgG antibody among participants from the two intervention communities was one-half that of the participants from the two non-intervention (no AGO traps) communities (22.9% vs. 45.4%; risk ratio = 0.52, 95% CI = 0.38–0.71). To date, this is the only evidence of a significant epidemiological impact by a mass-trapping intervention.

The Lorenzi et al. [58] report was preliminary, but the findings are centered on a multi-year mass-trapping intervention that significantly reduced adult female *Ae. aegypti* populations in the intervention areas. However, while these results are promising, the cost of the AGOs, which like the GAT are commercially available, is relatively high, likely dampening prospects for very large deployments. Of note, efforts to reduce costs by decreasing the number of traps deployed per house appear to result in decreased efficacy. A recent study in Clovis, CA, by Cornel et al. [59] using a single AGO trap per house (144 houses in total) failed to significantly reduce adult *Ae. aegypti* populations compared to the reductions experienced in the three traps/house interventions performed in Puerto Rico. However, no intensive source reduction was performed and the study’s scale was relatively small. One is hopeful that economies of scale as mass deployment of these larger lethal oviposition traps become more common will lead to significant reductions in trap prices.

## 4. The Need for Community Engagement and Participation

Reliance on vertically structured government programs (top–down) standard in yellow fever control in the early 20th century, and still common to dengue control programs in the 1980s, has since shifted to community-based (bottom–up) programs [60]. This practice started from the realization that disease control programs needed community engagement for long-term sustainability as well as due to diminishing government funds [61]. Although community-based interventions have generally failed to prevent epidemic dengue, there have been successes in mosquito control [62,63] and those willing to implement a mass-trapping intervention should consider adopting a community-based model.

At its most basic, this requirement is due to the colossal task of monitoring and servicing 100’s to 1000’s of traps, all of which, if neglected, can become productive larval habitats. Furthermore, for mass-trapping interventions to be a success, operators need continued access to the homes and yards of participants, as well as engaged and informed homeowners who recognize and alert operators to trap malfunctions. Ultimately, success through sustainability will rely on interagency cooperation and coordinated involvement of local health services, trained vector control personnel, and the community.

### Source Reduction Campaigns Minimize Competition from Alternative Oviposition Sites

Destruction or removal of alternative water-holding containers is particularly important for mass-trapping interventions targeting gravid *Aedes* females because they maximise the likelihood that the females will try to lay eggs first in the trap, killing them and all their progeny. However, source reduction for control of invasive *Ae. aegypti* and *Ae. albopictus* poses one of the greatest challenges for dengue and Zika control. The difficulties arise from the ability of both species to exploit small pockets of water, often inaccessible because they are located in private areas within the peridomestic environment [14,64,65]. These sources are also often ephemeral and their abundance increases and decreases on a continual basis [6]. Combined, these factors, as well as inadequate engagement of the local communities, likely accounted for the historical failures of traditional source reduction campaigns [66]. However, recent community-based control initiatives, emphasising source reduction and other non-insecticide methods of control, have been successful in Cuba [67], Singapore [68], Nicaragua and Mexico [69], as well as India [70]. The success of these programs can be largely attributed to extensive communication and cooperation among agencies, as well as the use of wide-scale education efforts (e.g., school visits, use of community volunteers, door-to-door demonstrations [21]). Of note, because they exploit and expose specific mosquito behavior, lethal oviposition traps can be useful tools for active learning, providing “teachable moments” and indelible hands-on experience that are worth thousands of written words (Fonseca, D.M., personal experience).

## 5. Recommendations, Conclusions and Future Directions

Based on the few available studies summarized and our own experience, we propose several recommendations to help achieve the greatest entomological and epidemiological impact. These recommendations are:

1. Conduct source reduction campaigns before implementing mass-trapping intervention to remove competing containers. These efforts should involve collaboration and coordination among all groups involved, especially the residents.
2. Involve homeowners in the maintenance/servicing of traps to achieve short-term success and long-term sustainability. Motivating homeowners to purchase their own traps, particularly in developed countries, and possibly at subsidized rates, may enhance participation in community-based mosquito control, including source reduction (step 1).
3. Aim for a minimum of >80% coverage (i.e., number of houses with traps) within intervention areas. Due to the limited data available, this is a “best guess” estimate based on the success of the AGO interventions (81%–85% coverage) in Puerto Rico [56,57], which resulted in a positive epidemiological outcome [58]. However, this value will likely depend on variables such as the type and size of the trap deployed and the density and type of housing in the intervention location. As such, further field trials are needed to determine the optimal coverage percentage across trap and location types.
4. Optimize the number of traps per yard based on the size of the property, trap size and placement, and number of competing water-holding containers. Again, undertaking source reduction campaigns, while unlikely to overcome the spatial variability in target populations created by cryptic breeding sites, will reduce and, to an extent, even out competition from secondary breeding sources across residences. This will help operators optimise trap interventions based on easy-to-assess metrics such as property and trap size, all the while helping to reduce the target population prior to the intervention.
5. Use large autocidal gravid traps, such as the AGO and the GAT, to maximise visual and olfactory attraction to achieve optimal suppression, while limiting the number of traps deployed. Economies of scale will inexorably bring the price of the traps down as more programs start buying them in bulk. To this point, initial investments and ongoing operational costs will need to match or reduce operational costs relative to traditional vector control strategies such as space spraying to be attractive to public health programs. The annual per-household costs of various dengue vector control interventions have been estimated to cost between $1.89 and $31.75 [71,72,73]. Although there is no available costing data for mass-trapping interventions, the long service intervals (2–4 weeks or longer) and ability to reuse the traps for multiple seasons, combined with emphasis on community involvement to reduce staffing, will likely help trap-based interventions to match or reduce these costs. However, further research is needed involving long-term interventions before cost estimates can be provided.
6. Avoid the use of organophosphate and synthetic pyrethroid insecticides, as insecticide resistance is increasing in urban *Aedes*. The use of edible canola oil makes GATs appealing to the public who find them “safe” to use (Fonseca, D.M., unpublished data [41]).
7. While the design of most lethal oviposition traps prevents adult emergence from dropped eggs from escaping, treat infusions with S-methoprene or long-release Bti formulations to further minimize the likelihood that traps may become productive larval habitats. Bti is particularly well suited for community-based interventions as many formulations are commercially available and do not require specialized applicator licenses or permits. These products can also be provided by a local public health agency and simply added to trap infusions at each service interval to maintain efficacy.
8. Use natural grass or hay infusions as the olfactory lure to attract gravid *Ae. aegypti*. The infusions can last for up to 2 months or longer (see Mackay [39] or Barrera [56]). This can be tasked to residents who can simply change water and add hay or other organic material provided by the control program. The infusion material can also be sourced locally from their own yards (e.g., dead leaves, grass clippings) at no cost. Of note, field surveys by Johnson et al. [74] suggest that aromatic infusions may be unnecessary as they found no difference in the number of gravid *Ae. albopictus* females captured in GATs baited with (hay or fish food) and without (water or empty) aromatic infusions. This finding is supported by Trexler et al. [75], who found that the initial female attraction to the trap may be overwhelmingly associated with the water rather than an olfactory attractant produced by the infusion. If extendable to other species and locations, the omission of aromatic infusions would greatly extend and simplify trap servicing, reducing costs and reliance on homeowners to maintain infusions, and possibly increasing participation by homeowners opposed to having “smelly” traps around the house. These observations undoubtedly warrant further investigation, but until more data is available, the recommendation remains for the inclusion of aromatic infusions.
9. Use a mapping strategy (e.g., GIS) during trap placement so traps can be easily found. This will be a useful tool to ensure that traps are accounted for even if a bottom–up approach is used in which residents are encouraged to purchase their own traps. For example, a serial number or barcode could be assigned to individual traps and reported to the corresponding public health program overseeing the intervention. This identifier could then be linked to the residential address, allowing the agency to periodically check to ensure that the traps are being properly maintained, as well as track missing traps.

Of the eight studies summarised involving mass deployment of lethal oviposition traps (Table 1), seven resulted in a significant reduction in adult female *Ae. aegypti* abundance in at least one intervention site, whereas five of these resulted in significant reductions across all sites. However, only two studies attempted to assess the epidemiological impact of the intervention and only one showed a significant reduction in disease prevalence. Based on these results, further large-scale randomised controlled trials in disease endemic countries are needed before mass-trapping interventions of lethal oviposition traps can be recommended as a means of suppressing disease. However, there seems to be sufficient supporting evidence to recommend their use as a means of suppressing female *Ae. aegypti* populations.

Furthermore, while to the best of our knowledge all mass deployments of lethal oviposition traps so far have been on *Ae. aegypti* in resource-poor or tropical settings (Table 1), we propose that mass deployment of lethal oviposition traps can be developed as a pre-emptive strategy for control of temperate *Ae. albopictus* in resource-rich communities, because: (1) mass deployments can be focused in a relatively short period of time in late spring to prevent exponential population growth to levels that allow disease transmission; (2) as mentioned, these traps are a gateway strategy to involve residents in local mosquito control, especially if they become directly “invested” by purchasing the traps themselves (a relatively small investment in medium- and high-income neighbourhoods); (3) by their simplicity and direct association to an understandable mosquito behaviour (oviposition) and control outcome (i.e., observable dead mosquitoes), lethal ovitraps are an excellent “teaching tool” that easily feeds back to community-level source reduction as residents understand the need to reduce competing water-holding containers. A win-win.

## Figures and Tables

**Table 1:** Summary of lethal oviposition trap mass-trapping interventions to control female *Aedes aegypti* populations.

Standard Lethal Ovitraps								
Author	General Trap Design	Killing Agent	Length of Intervention/Study Location	Number of Traps per Residence	Reduction Achieved	% Residences Covered	Other Interventions Involved	Epidemiological Outcome
Perich et al. [30]	Black 473 mL cup baited with 10% hay (w/v) infusion	11 × 2.5 cm ovistrip treated with deltamethrin	3 months; Areia Branca and Nilopolis, Rio de Janeiro, Brazil	10	Female adult abundance reduced 47% at one site; % containers positive for *Ae. aegypti* larvae reduced 49% and 80%; Mean pupae per house reduced 97% and 91%	Not specified	None	Did not measure
Sithiprasasna et al. [33]	Black 473 mL cup baited with 10% hay (w/v) infusion	11 × 2.5 cm ovistrip treated with deltamethrin	Two studies; each 12 months in length; Ratchaburi Province, Thailand	10	First study (1999): No reduction Second study (2000): 47% reduction in female adult abundance; 49% reduction in containers with *Ae. aegypti* larvae; 56% reduction in containers with *Ae. aegypti* pupae	Not specified	None	Did not measure
Rapley et al. [46]	1.2 L black bucket set with 1 L of water and a 0.5-g alfalfa pellet	13.5 × 5 cm ovistrip treated with bifenthrin	4 weeks/site; Cairns, Queensland, Australia	4	Wet season: 87% reduction in sticky ovitrap collections; reductions in BG-Sentinel (BGS) collections not specified	75% Dry season; 71% Wet season	Larval control: source reduction and treatment of potential breeding sites with S-methoprene	Did not measure
Rapley et al. [46]	Biodegradable ovitrap: 1.2 L volume set with 1 L of water and a 0.5-g alfalfa pellet	13.5 × 5 cm ovistrip treated with bifenthrin	4 weeks/site; Cairns, Queensland, Australia	4	Reduction observed in 1 out of 3 sites; % reduction not specified	93% Wet season	Larval control: source reduction and treatment of potential breeding sites with S-methoprene	Did not measure
Regis et al. [54]	Modified 2 L bottles painted black	*Bacillus thuringiensis israelensis* (Bti)-treated water; egg strips incinerated upon collection	24 months; Ipojuca and Santa Cruz do Capibaribe; Pernambuco, Brazil	5	90% and 77% in egg density in two separate study sites	Not specified; 8400 ovitraps installed during intervention	larvivorous fish and adult aspiration	Did not measure
**Sticky Ovitraps (adhesive sticky cards/panels)**								
Degener et al. [51]	MosquiTRAP: 700 mL black plastic cylinder filled with 300 mL water	Black adhesive card. Card contained AtrAedes^®^ oviposition attractant	17 months: Manaus, Amazonas, Brazil	3	No, observed an increase in trap counts in intervention sites	51.1%	None	No difference in dengue virus (DENV) IgM seropositivity between intervention and control sites
**Large (>5 L) Autocidal Gravid Ovitraps**								
Barrera et al. [56]	AGO: 19 L black bucket and 3.8 L black cylindrical entrance; baited with hay infusion (10 L water +10 g hay)	Black adhesive card placed on inside of trap entrance	12 months; La Margarita, Puerto Rico	3–4	53% reduction in BGS collections; 70% reduction in sentinel AGO traps	81%	Source reduction, larviciding and oviciding (physical destruction of eggs) prior to trap deployment	Did not measure
Barrera et al. [57]	AGO	Black adhesive card placed on inside of trap entrance	24 months; La Margarita, Puerto Rico 12 months; Villodas, Puerto Rico	3	La Margarita: 79% reduction in sentinel AGO collections; Villodas: 88% reduction in sentinel AGO collections	85%	Source reduction, larviciding and oviciding (physical destruction of eggs) prior to trap deployment	Did not measure
Lorenzi et al. [58]	AGO	Black adhesive card placed on inside of trap entrance	Continuation of study by Barrera et al. [57]: 1 year prior to introduction of chikungunya and CHIKV IgG serosurvey	3	Not specified, but same study areas as Barrera 2014a,b	85%	Same as Barrera et al. [56,57]	Yes, 52% reduction in chikungunya virus (CHIKV) IgG antibody prevalence in intervention areas (risk ratio = 0.52, 95% CI = 0.38–0.71). 62% of households and 64% of eligible participants surveyed.
Cornel et al. [59]	AGO	Black adhesive card placed on inside of trap entrance	6 weeks; Clovis, CA, USA	1	No, % reduction not specified. Small slopes of regression in weeks 3–8 in intervention site (BGS = −0.0047 and AGO = −0.0035) indicate reduction due to AGOs was minimal	Not specified; 144 residences in a single intervention area	None	Did not measure
